# A Cyber-ITS Framework for Massive Traffic Data Analysis Using Cyber Infrastructure

**DOI:** 10.1155/2013/462846

**Published:** 2013-05-16

**Authors:** Yingjie Xia, Jia Hu, Michael D. Fontaine

**Affiliations:** ^1^Hangzhou Institute of Service Engineering, Hangzhou Normal University, 222 Wenyi Road, Hangzhou 310012, China; ^2^Department of Civil and Environmental Engineering, University of Virginia, 351 McCormick Road, Charlottesville, VA 22903, USA

## Abstract

Traffic data is commonly collected from widely deployed sensors in urban areas. This brings up a new research topic, data-driven intelligent transportation systems (ITSs), which means to integrate heterogeneous traffic data from different kinds of sensors and apply it for ITS applications. This research, taking into consideration the significant increase in the amount of traffic data and the complexity of data analysis, focuses mainly on the challenge of solving data-intensive and computation-intensive problems. As a solution to the problems, this paper proposes a Cyber-ITS framework to perform data analysis on Cyber Infrastructure (CI), by nature parallel-computing hardware and software systems, in the context of ITS. The techniques of the framework include data representation, domain decomposition, resource allocation, and parallel processing. All these techniques are based on data-driven and application-oriented models and are organized as a component-and-workflow-based model in order to achieve technical interoperability and data reusability. A case study of the Cyber-ITS framework is presented later based on a traffic state estimation application that uses the fusion of massive Sydney Coordinated Adaptive Traffic System (SCATS) data and GPS data. The results prove that the Cyber-ITS-based implementation can achieve a high accuracy rate of traffic state estimation and provide a significant computational speedup for the data fusion by parallel computing.

## 1. Introduction

The quantity and quality of sensor data collected dedicatedly for transportation systems have increased tremendously in the past few decades. This trend will continue in the foreseeable future and leads to a new research direction on traffic data analysis in intelligent transportation systems (ITS). Intelligent data analysis, supported by mathematical algorithms, is becoming an important tool for scientific discovery and decision making in many fields, for example, bioinformatics, geographic information systems, and ecology. By integrating intelligent data analysis into ITS, a research direction named data-driven ITS emerges to focus on algorithmic and intelligent analysis on multisensor heterogeneous traffic data. This new direction leads to a revolution in ITS development, which adopts vision, multi-source, and learning-based traffic data processing algorithms for performance optimization [1]. In recent years, data-driven ITS is becoming more feasible in ITS since a variety of traffic sensors are widely deployed in the urban and suburban areas.

As data size and algorithmic complexity increase, data-driven ITS faces significant challenges in terms of solving computation-intensive problems. These problems lead to excessive lag in the execution time of the applications. A solution is proposed to migrate computation-intensive ITS applications from the conventional single-CPU architecture to the emerging Cyber Infrastructure (CI). CI is by nature a parallel-computing architecture and software environment, which is urgently needed by applications that must be run in real time.

In this paper, a new Cyber ITS framework is proposed to synergistically integrate data analysis algorithms into ITS and realize efficient processing of massive traffic data sets using CI. This framework is urgently needed for the following reasons: (i) ITS demands transformative breakthroughs to solve computationally intensive data processing problems; (ii) CI is now increasingly accessible to ITS and data analysis [2]; and (iii) some theoretical investigation on using CI for ITS has been conducted [3]. Cyber-ITS is technically designed to provide a traffic data and operation sharing framework based on parallel computing techniques. The framework is also designed following the component-and-workflow-based model, which enables different applications to assemble their relevant functionality components into workflows.

The rest of the paper is organized as follows. The next section reviews the past research relating data-driven ITS and CI. The design of the new Cyber-ITS framework is then introduced, and a case study of traffic state estimation using parallelized traffic data fusion is presented. Afterwards, the accuracy and efficiency tests are conducted on the studied case. Finally, conclusions and recommendations for future research are discussed.

## 2. Related Work

Conventional ITS is evolving into data-driven ITS, which focuses on processing data collected from multiple traffic sensors. Depending on the types of data and the fashion that the data is processed, the data-driven ITS is categorized into three types: vision-driven ITS, multisource-driven ITS, and learning-driven ITS. Vision-driven ITS takes video cameras as traffic sensors, which collect data for some representative applications, such as traffic object detection [4] and recognition [5], traffic behavior analysis [6], and vehicle trajectory construction [7]. However, vision-driven ITS suffers from some constraints, for example, weather, brightness, and camera positions. Therefore, to overcome the weakness of vision-driven ITS, multisource-driven ITS proposes to use multiple types of sensors, so that different kinds of sensor, can compensate each other's weaknesses. For example, the loop detectors deployed in-pavement are robust to environmental constraints, but have the drawbacks of a high failure ratio and potential inaccurate calculation of traffic state [8]. On the contrary, probe vehicles (e.g. GPS data) can provide true point-to-point travel times, but small sample size could result in poor statistical representation, and probes could be subject to errors in the map-matching process [9]. Multisource-driven ITS fuses heterogeneous data from video sensors, loop detectors, and probe vehicles to improve the accuracy and completeness of traffic data [10]. No matter what kinds of traffic sensors are used, it is always required to have the corresponding learning-based analytical tools to process data. This leads to the learning-driven ITS. Some commonly used learning algorithms, including rough set theory [11], online learning [12], fuzzy logic [13], are used to extract intrinsic rules from historical and real-time traffic data. These kinds of work always run into challenges of computational efficiency when dealing with large data sets. Luckily, this problem can be effectively solved by algorithmic parallelization executed on CI resources [14].

CI has been widely adopted in computer systems for its improved computing capability and cost effectiveness. Multinode, many-core, and general purpose graphics processing unit (GPGPU) are three major architectures of CI. Above the underlying hardware, the application-oriented techniques, such as data decomposition [15] to generate multiple computing tasks, task scheduling to allocate computing nodes, nodes communication for tasks interaction, and computation synchronization [16] for results collection are leveraged to support computation-intensive and data-intensive problems solving. These techniques are integrated as a piece of middleware, which delivers interoperable and reusable services for efficient use of CI resources [17].

Recently, some research on the combination of data-driven ITS and CI has been published, such as parallel implementation of transportation network models [18], computationally efficient online identification of traffic control intervention measures [19], parallel traffic control and management [20], and Message Passing Interface (MPI)-based parallelized fusion for traffic state estimation [21]. However, these studies are just for specific applications and cannot be extended to build a generic framework. Nevertheless, current applications all show the need for data sharing and applications integration. Therefore, this research seeks to develop Cyber ITS as a framework that can perform heterogeneous data analysis on CI in the context of ITS.

## 3. Cyber-ITS Framework

**Figure 1:**
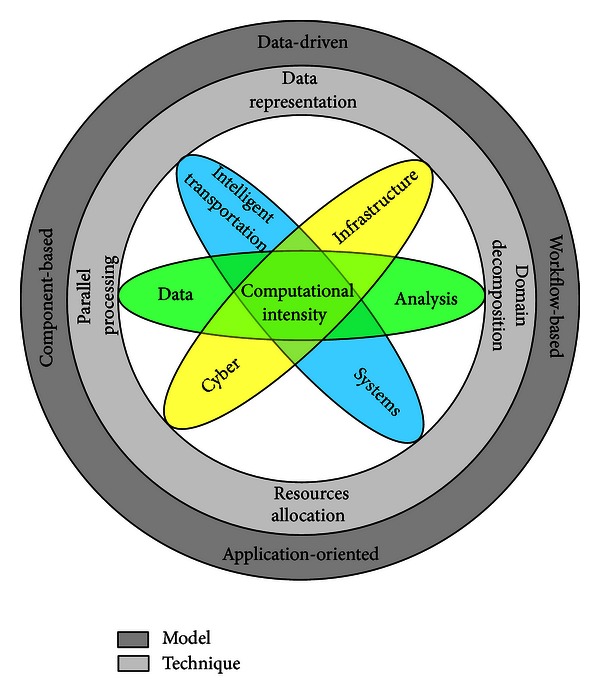
The Cyber-ITS framework synthesizes CI and data analysis in the context of ITS.

The Cyber-ITS framework is designed to synthesize CI and data analysis in the context of ITS, as shown in Figure 1. These three fields are interlinked on computational intensity, which plays a central role in the framework. On the technique level, a collection of technical tools are equipped to complete the tasks of data representation, domain decomposition, resources allocation, and parallel processing based on the computational intensity. These tools, such as ITS computational domain, octree-based decomposition, and max-min tasks scheduling, can improve the computing performance of traffic data analysis by parallel computing. The techniques are organized as the models on the outside layer in Figure 1, such as data-driven, application-oriented, and component-and-workflow-based models. The details about computational intensity, techniques, and models of the Cyber-ITS framework are specified as follows.

### 3.1. Computational Intensity

Computational intensity is fundamental to the Cyber-ITS framework. The definition of computational intensity is derived from the traditional computational complexity theory, which focuses on assessing the algorithmic complexity in theoretical computer science [22]. However, the complexity theory only evaluates the algorithmic structure without adequately capturing the spatiotemporal characteristics of ITS data analysis. These characteristics, such as spatiotemporal clustering, neighborhood, autocorrelation, and interaction dynamics of massive traffic data, need to be considered in the transformed computational intensity and therefore can reinforce the algorithmic complexity in evaluating computing time, memory, and input/output (I/O).

Again, computational intensity is the most important measurement in this framework. Supposing a computation-intensive ITS application needs to be completed in real time, it can be parallelized to execute on CI. The parallelization work first requires the application to be divided into multiple computing tasks. Since the efficiency of parallel computing is determined by the agreement between tasks loads and CI computing capabilities, the computational intensity is regarded as the right measurement that can quantify tasks loads during the task division. The details about the computational intensity will be specified in a case study later in this paper.

### 3.2. Techniques

#### 3.2.1. Data Representation

**Figure 2:**
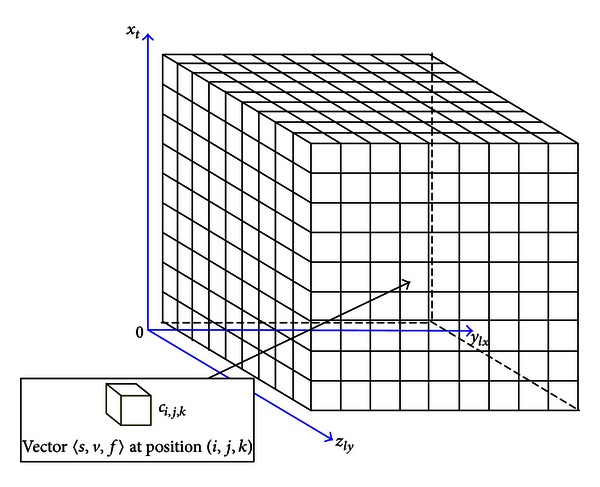
ITS computational domain projects heterogeneous traffic data into three-dimensional space.

Before addressing computational intensity, the explicit data representation which takes into account the characteristics of data and operations needs to be investigated. The Cyber-ITS framework adopts an ITS computational domain theory to formally represent heterogeneous data collected from multiple types of traffic sensors. ITS computational domain, which is derived from the idea of spatial computational domain [23], can project different types of traffic data from a high-dimensional data space to a three-dimensional spatiotemporal domain *D* = (*c*_*i*,*j*,*k*_) consisting of a large amount of cells. For example, as shown in Figure 2, to represent traffic data from Sydney Coordinated Adaptive Traffic System (SCATS) detector loops and GPS probe vehicles, the cell *c*_*i*,*j*,*k*_ can be defined as a vector <*s*, *v*, *f*> at position (*i*, *j*, *k*). *s* denotes the sensor type which has two values: 0 and 1, 0 standing for SCATS and 1 standing for GPS. *v* and *f* denote the speed and flow of a road segment, respectively. The coordinates (*i*, *j*, *k*) indicate the discretized time and location indices of a road segment in the respective *x*_*t*_, *y*_*lx*_, and *z*_*ly*_ axes.

#### 3.2.2. Domain Decomposition

**Figure 3:**
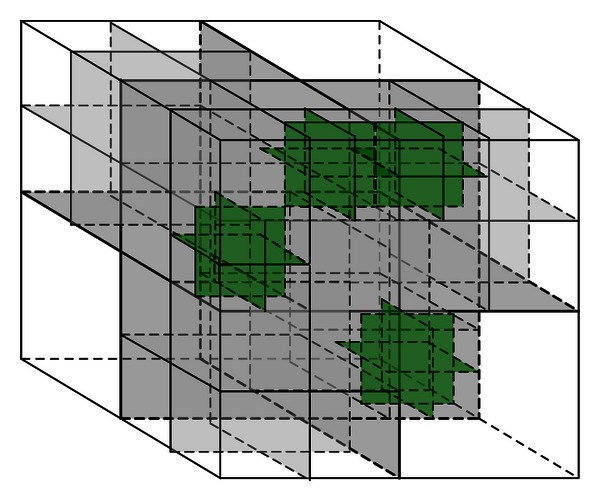
ITS computational domain is decomposed by octree.

When an application using data in the ITS computational domain requires CI resources for parallel computing, the domain first needs to be decomposed into subdomains to create multiple computing tasks. To maximize the efficiency of CI recourses, it is necessary to assure that the work loads are balanced among subdomains. The load balance of these subdomains can be achieved by using a technique called octree [24]. The octree would recursively divide a domain or subdomain into eight smaller subdomains until certain criterion on computational intensity achieving a threshold is met. In the octree-based ITS computational domain decomposition, computing time, memory, I/O, or their combination are the candidate criteria. The threshold is determined by dividing the computational intensity of the whole computational domain by the maximum number of CI computing nodes. A demonstration of octree-based domain decomposition is shown in Figure 3.

#### 3.2.3. Resource Allocation and Parallel Processing

The subdomains generated from octree-based domain decomposition enable multiple computing tasks to be created from one single data-driven ITS application. These subdomains are then assigned to CI resources. As one of the most popular CI resources, a cluster computer consisting of a set of loosely connected computing nodes is commonly designed as a homogeneous architecture. Therefore, in the cluster computer the parallel processing on ITS computational domain can be implemented by simply allocating computing nodes to computing tasks on subdomains one by one. If the number of tasks is greater than the number of nodes, some algorithms are required to make the computing tasks scheduled to each node as load balanced as possible. For example, the max-min scheduling algorithm [25] combines a high-loaded task with a low-loaded task as one task package scheduled to one cluster node. This algorithm can avoid generating the weakest link in a chain of computational intensity assigned to each computing node and improve the efficiency of parallel processing in the Cyber-ITS framework.

### 3.3. Models

#### 3.3.1. Data-Driven and Application-Oriented Models

**Figure 4:**
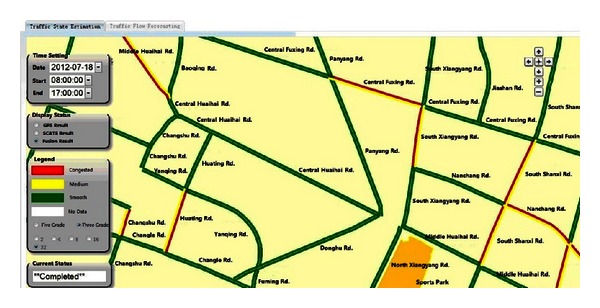
A common portal system serves for Cyber-ITS-based traffic state estimation and traffic flow forecasting.

The aforementioned techniques of the Cyber-ITS framework are used in data-driven and application-oriented models. Diverse data-driven ITS applications share the same formally represented traffic data and utilize the corresponding methodology to process the data. For example, traffic state estimation [2] and real-time traffic flow forecasting [26] are two Cyber-ITS-based applications on ITS computational domain utilizing SCATS and GPS data. For traffic state estimation, the fusion of heterogeneous traffic data is adopted to combine the strength of different traffic sensors and compensates for their corresponding deficiencies. The fusion process consists of four steps: data smoothing, traffic state features estimation, features fusion, and decision making [3]. All four steps are involved with the data processing on the ITS computational domain. For real-time traffic flow forecasting, SCATS and GPS data are first transformed to estimate the flow as the historical data. These data construct the covariance data matrix, which is used in the modal functions of spectral analysis. This method can predict the traffic flow of 1 hour and 15 minutes in the future within 15 minutes. The process of traffic flow forecasting consists of three steps: traffic flow estimation, covariance matrix construction, and online prediction by spectral analysis [26]. Similar to the previous traffic state estimation, all these three steps relate to the data processing on ITS computational domain. A common portal system of these two Cyber-ITS-based applications is shown in Figure 4. The portal system can provide basic input and output interfaces for the applications.

#### 3.3.2. Component-and-Workflow-Based Model

Diverse data-driven ITS applications not only share common formally represented data but also reuse several processing modules, for example, the features estimation in both traffic state estimation and traffic flow forecasting. The Cyber-ITS framework adopts a component-based model to encapsulate all processing modules as components. Some commonly used components are categorized as: (i) high-performance computing components encompassing CI-based parallel computing for ITS and data analysis; (ii) data-related components interacting with high-performance computing components to evaluate the computational intensity and process massive traffic data; (iii) collaboration components provide collaborative support for interactions management among different applications. These components can guide the implementation of data-driven ITS applications based on the Cyber-ITS framework.

The interoperable and reusable components can compose workflow to develop Cyber-ITS-based applications [27]. The workflow is based on flexible and scalable composition of individual components following common interfaces and standards [28]. For the example of traffic state estimation, the workflow is composed of high-performance computing components and data-related components to realize the capabilities of high-performance computing, traffic data processing, and features fusion. These components also need to be configured and managed in the specific contexts of ITS applications.

## 4. Case Study in Traffic State Estimation

### 4.1. Introduction

**Figure 5:**
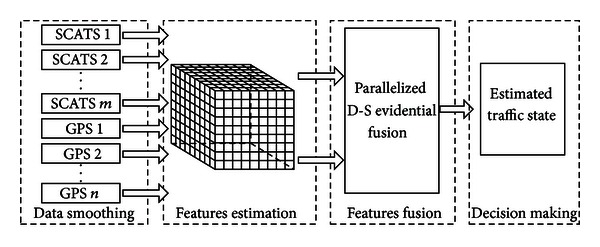
Flowchart of traffic state estimation consists of data smoothing, features estimation, features fusion, and decision making.

One typical data-driven ITS application, traffic state estimation by fusing SCATS and GPS data, is chosen as a case to study the implementation of the Cyber-ITS framework. The aforementioned four steps of traffic state estimation are flowcharted in Figure 5.

The data smoothing is first performed using Kalman filters to reduce the impact of noise on the collected data [9]. Then, the estimation algorithms are adopted to transform the filtered SCATS and GPS source data into traffic state features [14], such as speed and flow rate. These features are used to construct the ITS computational domain. Afterwards, the features from different kinds of sensors on one road segment are fused by Dempster-Shafer (D-S) evidence theory [29], which can overcome the conflict of estimated traffic states from different sensors. D-S evidence theory is a mathematical theory mostly used when combining evidence from different sources. The theory uses the belief function to express the probabilities of various combinations of evidence as degrees of belief. In traffic state estimation, the evidence is just the discretized values of traffic state estimated by different sensors, such as “congested,” “medium,” and “smooth.” The algorithm of D-S evidential fusion on the discretized traffic state of a cell in the computational domain is shown as follows:

(1) m(st)=m1(s1,t)⊕m2(s2,t)⋯⊕mX(sX,t)=∑⋂i=1Xsi,t=st(∏i=1Xmi(si,t))1−∑⋂i=1Xsi,t=Φ(∏i=1Xmi(si,t)),

(2) $$\begin{array}{c} m_{i}\left(s_{i,t}\right)=\frac{n_{i,t}\left(s,d\right)}{n_{i,t}\left(d\right)}, \end{array}$$

where *m*(*s*_*t*_) denotes basic probability assignment (BPA) of the specified traffic state *s* as the fusion result at time *t*, and *m*_*i*_(*s*_*i*,*t*_), *i* = 1,2,…, *X* represents the BPA of this state from *i*th sensor at time *t*. The *m*_*i*_(*s*_*i*,*t*_) of one cell can be estimated by calculating the proportion which divides the number of its neighboring cells *n*_*i*,*t*_(*s*, *d*) within a specified distance *d* and having that state *s* by the number of all neighboring cells within *d*. Traffic state is determined following a typical decision rule in D-S evidence theory, maximum degree of belief [30].

### 4.2. Parallelized Fusion

As the amount of SCATS and GPS data is relatively high in the context of ITS applications, it is a great challenge to complete the data fusion on a real-time basis. In this study, data are collected from downtown Shanghai, where 2077 road segments are included. The GPS data are gathered from 8172 taxis belonging to Jinjiang and Bashi taxi companies, and the SCATS data are from 1836 loop detectors deployed on around 200 road intersections. All the collected data are uploaded to the server once every 2 minutes. The total number of SCATS and GPS data records during 10 minutes exceeds 50000; hence, it would take more than one hour to execute the aforementioned four steps of fusing traffic features by sequential computing. However, the real-time nature of the computation requires the calculation to take less than 10 minutes. Therefore, as a solution, data-centric parallelization of D-S evidential fusion is proposed to improve computing performance.

The parallelization process consists of (i) evaluating the computational intensity of data fusion by calculating CPU cycles; (ii) octree-based decomposition of the computational domain into subdomains according to the CPU cycle threshold; and (iii) scheduling subdomains to the computing nodes of a cluster computer for parallelized D-S evidential fusion. Specifically, as the computational intensity evaluation, the CPU cycles of data fusion on one road segment are counted as follows:

(3) NCPUCycles=BPAnumber×Nneighbors+BPAnumber×(2×BPAnumber2+2×BPAnumber2),

where *BPA*_number_ × *N*_neighbors_ indicates the number of CPU cycles for calculating the BPA values for all traffic states in (2), and two 2 × *BPA*_number^2^_, respectively, indicate the number of CPU cycles of ∑_⋂_*i*=1_^*X*^*s*_*i*,*t*_=*s*_*t*__(∏_*i*=1_^*X*^ *m*_*i*_(*s*_*i*,*t*_)) and ∑_⋂_*i*=1_^*X*^*s*_*i*,*t*_=Φ_(∏_*i*=1_^*X*^ *m*_*i*_(*s*_*i*,*t*_)) in (1). The threshold is determined by dividing the number of CPU cycles of the whole computational domain by the number of cluster nodes [31], and the tasks scheduling is conducted using the max-min scheduling algorithm if the number of subdomains is greater than the number of computing nodes. In the above example, the CPU cycles of data fusion on one road segment are counted as follows:

(4) NCPUCycles=BPAnumber×Nneighbors+BPAnumber×(2×BPAnumber2+2×BPAnumber2)=3×Nneighbors+108,

where *BPA*_number_ is equal to 3 standing for three values of discretized traffic states. Supposing that the number of neighboring cells *N*_neighbors_ is set as 100, for all 2077 road segments, *N*_CPU_Cycles__ is calculated as 847416. The corresponding threshold *thr* is determined by

(5) $$\begin{array}{c} thr=\frac{847416}{N_{\text{nodes}}}, \end{array}$$

where *N*_nodes_ denotes the maximum number of computing nodes. The threshold is further used to conduct the domain decomposition.

### 4.3. Cyber-ITS-Based Implementation

The traffic state estimation is implemented fully based on the Cyber-ITS framework. Massive SCATS and GPS data are fused using the techniques including ITS computational domain, octree-based domain decomposition, max-min-based subdomains scheduling, and parallelized fusion, which, respectively, correspond to data representation, domain decomposition, resources allocation, and parallel processing in the technique level of Cyber ITS. In the model level, this implementation also follows the SCATS-and-GPS-data-driven and traffic-state-estimation-oriented model. The computational domain, domain decomposition, and task scheduling are all reusable components, which can be shared among diverse data-driven ITS applications. These reusable components are combined with other specialized data processing components to construct workflow for parallelized fusion.

## 5. Experimental Results and Analysis

### 5.1. Experiment Setup

The experiments on the Cyber-ITS-based traffic state estimation are conducted on a Dawning TC4000L cluster which includes 64 computing nodes equipped with Intel Xeon Dual Core 2.4 GHz CPU and 2G memory. The SCATS and GPS data are uploaded by loop detectors and taxis, respectively, every 2 minutes from 8:00 to 17:00 on the chosen 393 road segments in downtown Shanghai. The data gathered per 10 minutes are calculated as one unit. The speeds of road segments estimated from SCATS and GPS data are formally represented as a computational domain. The computational intensity of data fusion on the whole ITS computational domain is evaluated as 160344 CPU cycles. The threshold is calculated as 160344/64 = 2505.4. Therefore, the computational domain is decomposed into 71 subdomains by octree. Both accuracy and efficiency tests of speed estimation by parallelized fusion are carried out on 1, 2, 4, 8, 16, and 32 computing nodes respectively. The speed can be discretized into “congested” (<5 km/h), “medium” (5 km/h–25 km/h), and “smooth” (>25 km/h). The accuracy of the fused traffic state is compared with a ground truth developed by manually analyzing available traffic video streams. From the captured snapshots of road intersections in the video, if all the vehicles waiting in the queue of a road segment cannot pass the intersection during one period of green light, we regard the traffic state of the road segment as “congested.” If all the vehicles can pass the road intersection during a half period of green light, we regard the traffic state as “smooth.” Otherwise, we regard it as “medium”. This level of comparison is analogous to red/yellow/green traffic condition maps commonly provided on internet traveler information websites by transportation agencies.

### 5.2. Results and Analysis

#### 5.2.1. Accuracy Test

**Figure 6:**
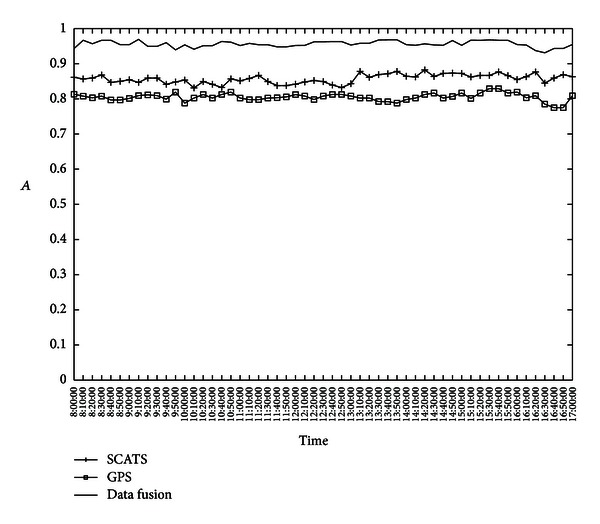
Accuracy rate of traffic state estimation is tested using SCATS data, GPS data, and their fusion.

The accuracy test uses a metric defined as follows:

(6) $$\begin{array}{c} A=\frac{n_{c}}{n_{\text{all}}}, \end{array}$$

where *A* denotes the accuracy rate of traffic state estimation, *n*_*c*_ is the number of correctly estimated states, and *n*_all_ is the number of states of all road segments in the whole time range. *n*_*c*_ is counted by comparing the discretized speed estimated from the sensors with the ground truth traffic state estimated from videos. The test cases include the traffic state estimation using SCATS data, GPS data, and their fusion. In the case that the traffic state of a road segment cannot be estimated due to sensor malfunctions or missing data, we regard the result as incorrect estimation. The experimental results on *A* of three test cases are shown in Figure 6.

As shown in Figure 6, the estimated traffic state using SCATS data is more accurate than that using GPS data. This is mainly caused by the smaller number of available GPS taxi probes in the sampling, which causes gaps in the network monitored by the probe vehicles. The average *A* of data fusion is around 0.95, which is higher than that of either SCATS or GPS. This is because of the synergistic effect of different types of traffic sensors, which can mutually compensate the loss of accuracy or data availability for each sensor. This accuracy test shows that the Cyber ITS framework can keep data availability and accuracy of the original application algorithms.

#### 5.2.2. Efficiency Test

**Figure 7:**
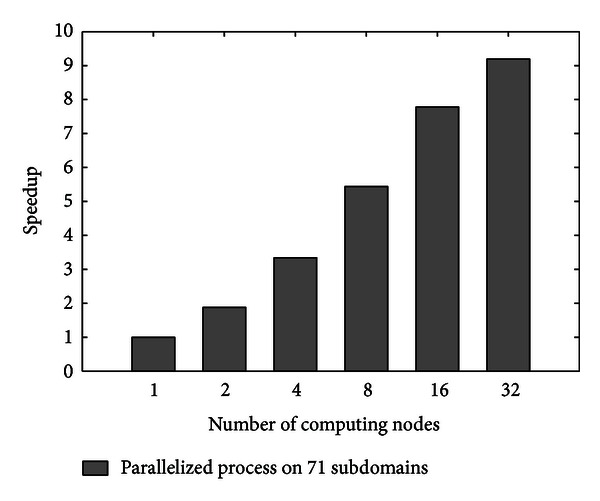
Speedup of parallelized process is tested on 1, 2, 4, 8, 16, and 32 computing nodes.

The efficiency test uses speedup as the metric to evaluate the acceleration performance of Cyber ITS. This metric is defined as a ratio of sequential execution time over parallel execution time in the following equation:

(7) $$\begin{array}{c} S=\frac{T_{1}}{T_{n}}, \end{array}$$

where *S* denotes the speedup, *T*_1_ is the sequential execution time using one computing node, and *T*_*n*_ is the parallel execution time using *n* computing nodes. The experiment evaluates the computing performance of 10-minute data processing, which consists of domain decomposition, tasks scheduling, and parallelized fusion. Average execution time is calculated by running the whole process on different number of computing nodes, as listed in Table 1. The results of speedup are also listed in Table 1, and illustrated in Figure 7.

As shown in Figure 7, as the number of computing nodes increases from 1 to 32, the speedup increases significantly. Clearly, the parallelized process allows data fusion to occur to support real-time traffic state estimation. When only one computing node is used, the average execution time exceeds 15 minutes, which is too long to support real-time applications. When 32 computing nodes are used, the average execution time decreases to less than 2 minutes. However, it is also found that the speedup increases slower than the increment of computing nodes. For example, when the number of computing nodes increases to 32, the speedup only reaches 9.19. This nonlinear trend is caused by the sequential execution components, such as domain decomposition and tasks scheduling. These components are also regarded as the overhead part in the parallelization. In addition, assuming fixed overhead time, as more computing nodes are used, the sequential execution components bring more impact on speedup of the whole process. Nevertheless, this efficiency test clearly shows that the Cyber-ITS-based implementation can perform significant speedup by parallel computing.

## 6. Conclusions and Future Work

The general goal of this paper is to propose a Cyber-ITS framework for the synthesis of CI, ITS, and data analysis. The development of the framework is motivated by the challenge of solving computation-intensive problems on processing massive and heterogeneous traffic data in real time. The Cyber-ITS framework presents a way to transfer the data-driven ITS applications from single-CPU architectures to parallel computing architectures. This transfer requires the transformation of applications, which are based on a common design for processing massive traffic data on CI.

As the core component of Cyber-ITS, the computational intensity is used to efficiently decompose the data represented by ITS computational domain into load-balanced subdomains. Besides the computational domain and domain decomposition, the Cyber-ITS framework adopts two other techniques, CI resources allocation and parallel processing, to complete the efficient parallelization on traffic data processing. All four techniques are synergistically integrated to follow both data-driven and application-oriented models and can build a component-and-workflow-based model to interoperate and reuse functional components among diverse data-driven ITS applications.

An example of traffic state estimation by fusing SCATS and GPS data was presented to test the effectiveness of the proposed Cyber-ITS framework. The example was implemented by using parallelized D-S evidential fusion, which consists of evaluating the computational intensity in CPU cycles, octree-based domain decomposition based on the computational intensity, and max-min-based tasks scheduling for parallelized fusion. The fusion work used the discretized traffic state estimated from different sensors and calculated the probabilities of their combination for final determination. This implementation fully followed the SCATS-and-GPS-data-driven and traffic-state-estimation-oriented models.

Traffic state estimation was also examined to test the performance of the Cyber-ITS framework. Two performance metrics, accuracy rate and speedup, were defined to conduct the respective accuracy and efficiency tests. The experimental results show that the Cyber-ITS-based data fusion achieved higher accuracy rate in estimating traffic state than using any single type of traffic sensor. In addition, as more computing nodes are employed, the experiment demonstrated that the Cyber-ITS framework can facilitate significant computational speedup by parallel computing.

In future work, the research team will design a piece of scheduling middleware for the purpose of dynamically adapting to different types of CI resources employed in the Cyber-ITS framework. Further research will also focus on expanding the implementation of data-driven ITS applications based on the Cyber-ITS framework. Moreover, the common framework portal system could be improved to gain more interoperability and reusability among diverse applications. 

## Figures and Tables

**Table 1:** Average execution time and speedup using 1, 2, 4, 8, 16, and 32 computing nodes.

Number of computing nodes	Average execution time/seconds	Speedup
1	927.6	1
2	492.9	1.88
4	277.3	3.34
8	170.6	5.44
16	119.2	7.78
32	100.9	9.19
